# Case report: An autopsy case of pilsicainide poisoning

**DOI:** 10.3389/fphar.2023.1090265

**Published:** 2023-01-26

**Authors:** Sella Takei, Hiroshi Kinoshita, Mitsuru Kumihashi, Mostofa Jamal, Hiroko Abe, Shoji Kimura

**Affiliations:** ^1^ Department of Forensic Medicine Faculty of Medicine, Kagawa University, Kagawa, Japan; ^2^ Bio Design Inc., Tokyo, Japan

**Keywords:** pilsicainide, poisoning, pro-arrhythmia, anti-arrhythmic agent, autopsy

## Abstract

We present a fatal case of pilsicainide poisoning. Quantitative toxicological analysis revealed that the concentrations of pilsicainide in femoral blood and urine samples were 17.5 μg/mL and 136.9 μg/mL, respectively. No morphological changes due to poisoning were observed. Based on the autopsy findings, results of the toxicological examination, and investigation by the authorities, we concluded that the cause of death was due to pilsicainide poisoning.

## 1 Introduction

Pilsicainide, a class IC anti-arrythmic agent according to the Vaughan Williams classification, is prescribed for the treatment of supraventricular and ventricular tachyarrhythmia (Plosker, 2010; Baselt, 2017a) and is available in Japan and Korea (Plosker, 2010). It is rapidly absorbed from the gastrointestinal tract following oral administration, the elimination half-life is 4.4–4.9 h following single oral administration, and the volume of distribution (Vd) is 1.48 L/kg (Plosker, 2010). Its major electrophysiological action is a selective sodium channel blockade without effects on potassium channels, calcium channels, or adrenal receptors, causing a decrease in intracardiac conduction velocity and negative inotropic effects (Plosker, 2010). Severe intoxication (Ozeki et al., 1999; Horita et al., 2004; Nakata et al., 2006; Oe et al., 2009; Imazu et al., 2017; Oshima et al., 2019; Asano et al., 2020) and fatalities (Hikiji et al., 2008; Fukasawa et al., 2018) have been reported. Acute poisoning from cardiovascular drugs is mostly due to β-adrenergic antagonists or calcium channel blockers, and poisoning from anti-arrhythmic agents is relatively rare (Vucinić et al., 2003; Gummin et al., 2018). Here, we report a fatal case of poisoning by pilsicainide.

**FIGURE 1:**
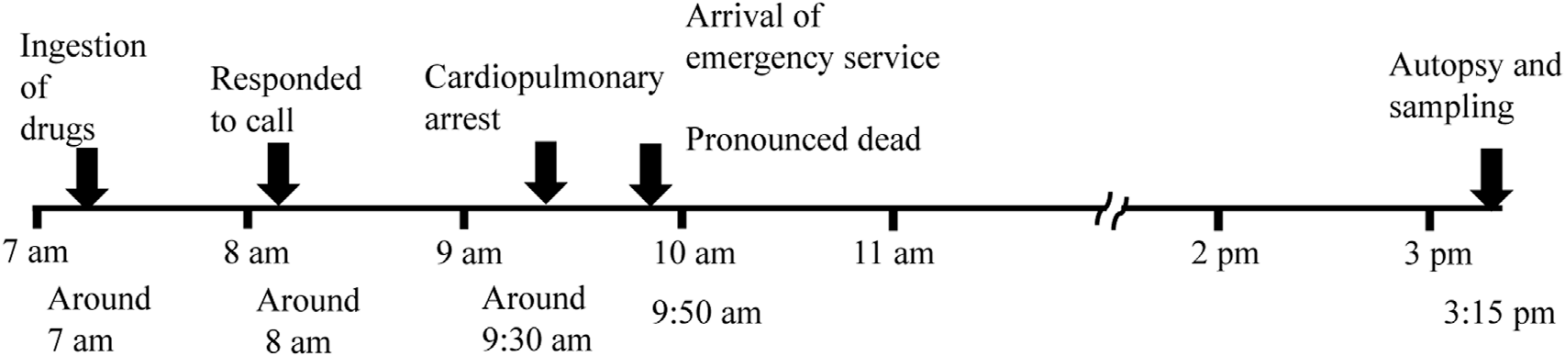
Timeline of the case.

A Japanese woman (78 years of age; height, 141 cm; and weight, 30 kg) was found dead in her house. She had been prescribed drugs (furosemide: 40 mg/day, pilsicainide: 150 mg/day, verapamil: 40 mg/day, and warfarin: 1 mg/day) for the treatment of arrhythmia and chronic cardiac failure. Her build was small for that of a Japanese, but she had no history of an eating disorder (BMI, 15.1). Her son saw her taking the drugs around 7 a.m. on the day of her death. Then, around 9:30 a.m., her husband noticed that she suffered cardiopulmonary arrest and called an ambulance. The ambulance arrived at 9:50 a.m.; however, as rigor mortis of the jaw joint was recognized, cardiopulmonary resuscitation was not attempted, and she was pronounced dead. The timeline of the present case is shown in Figure 1.

Autopsy findings indicated no evidence of external injury. The heart weighed 260 g and contained 330 mL of blood with a coagulum and a chicken fat clot. Histological examination revealed moderate fibrosis of the myocardium. The brain weighed 991 g and was atrophic, without injuries. The left and right lungs weighed 153 g and 200 g, respectively. The stomach contained a very small amount of reddish brown mucus. Signs other than congestion were not noted in other organs. A drug screening test using an IVeX Screen ^®^ M-1 (Biodesign Inc, Tokyo, Japan) panel resulted negative. Samples of postmortem blood (blood in the left and right heart chambers and femoral venous blood), urine, bile, cerebrospinal fluid, and stomach contents were collected for toxicological investigation.

Sample preparation for toxicological examination was as follows: D5-diazepam and D5-phenobarbital were added to 100 µL sample as an internal standard (IS) before adding 500 µL of acetonitrile. Stomach contents and bile and urine samples were also diluted 10 times with ultrapure water, and IS and acetonitrile were added, respectively, and extracted similar to the blood samples. Extraction was performed following vortex agitation, and the centrifuged supernatant of each extract was used for liquid chromatography with tandem mass spectrometry (LC-MS/MS) analysis.

Toxicological analysis using LC-MS/MS was performed as described previously (Kinoshita et al., 2017). Briefly, separations were carried out using ekspert™ ultraLC 100-XL (Eksigent Part of Sciex, Framingham, MA). An L-column2 ODS (1.5 mm × 150 mm, 5.0 µm particle size; Chemicals Evaluation and Research Institutes, Tokyo, Japan) was used with a mobile phase of solvent A (5% methanol containing 10 mM ammonium formate) and solvent B (95% methanol containing 10 mM ammonium formate) with a flow rate of 0.1 mL/min. A QTrap^®^ 4500 tandem mass spectrometer (Sciex) was used. Pilsicainide was detected by the electrospray ionization-positive mode using selective reaction monitoring (SRM). Quantitation of ethanol was performed using headspace gas chromatography.

## 2 Results and discussion

**FIGURE 2:**
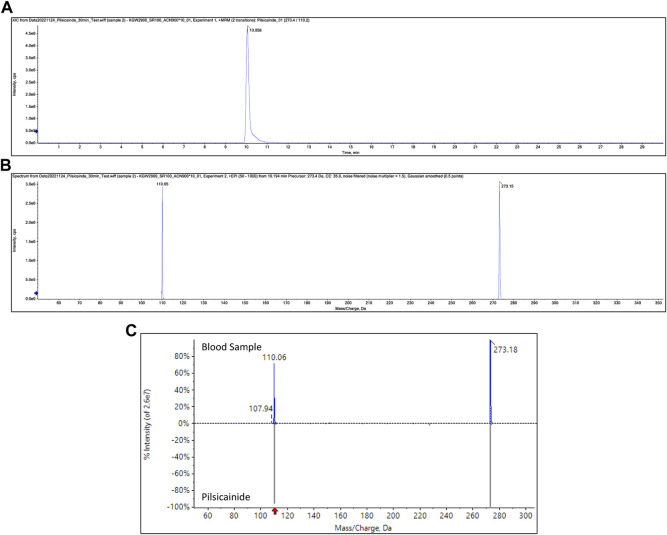
SRM chromatogram of pilsicainide in blood samples **(A)**, product ion spectrum of precursor ion m/z 273.15 at a retention time of 10.1 min **(B)**, and mass spectrum obtained from the blood sample and the mass spectrum of pilsicainide **(C)**.

Pilsicainide, verapamil, furosemide, warfarin, acetaminophen, ephedrine, and methylephedrine were identified in each sample through toxicological analysis. Figures 2A–C show the SRM chromatogram and mass spectrum of pilsicainide in the present case. Table 1 shows the concentrations in the postmortem samples, along with the currently established lethal, toxic, and therapeutic ranges (Schulz et al., 2020). No ethanol was detected in the postmortem samples.

**TABLE 1:** Concentrations of each drug in the post-mortem samples (µg/mL).

Specimen	Blood			Urine	Cerebrospinal fluid	Bile	Stomach contents		Therapeutic range^a^	Toxic range^a^	Lethal range^a^
	Femoral venous vein	Right heart chamber	Left heart chamber								
Pilsicainide	17.5	28.9	26.6	136.9	6.1	56.8	221.7		0.2–0.9	-	-
Verapamil	0.97	0.82	0.24	0.08	0.04	3.56	104.9		0.01–0.4	1	0.9–85
Furosemide	0.80	0.74	0.51	9.93	-	1.03	385.5		2–5	25–30	
Warfarin	0.70	0.63	0.25	0.05	0.02	0.25	0.08		1–7	10–12	100
Acetaminophen	B.D.L	B.D.L	B.D.L	0.04	B.D.L	B.D.L	B.D.L		10–25	100–150	200–300
Ephedrine	0.13	0.10	0.08	5.97	0.10	1.02	0.60		0.02–0.2	1	3.5–21
Methylephedrine	0.02	B.D.L	B.D.L	0.30	0.01	0.12	0.08		-	-	-

^a^ Therapeutic and lethal ranges are cited from Schulz et al. (2020).

B.D.L, below the detection limit.

Heart-to-peripheral blood concentration ratios of pilsicainide were within a range of 1.52–1.65 in the present case. This suggested a smaller postmortem distribution than that of another class IC anti-arrhythmic agent, flecainide (O’Sullivan et al., 1995). This finding may be due to the small Vd of pilsicainide (1.48 L/kg (Plosker, 2010)) compared to that of flecainide (5.5 L/kg (Tjandra-Maga et al., 1986; Hilberg et al., 1999)).

Following oral administration, 75%–86% of the pilsicainide dose is excreted through urine in an unchanged form, and a small proportion (4.5%–6.5%) of the dose is metabolized to 2-hydroxymethyate by cytochrome P450 (CYP) 2D6 (Fujitani et al., 1997) and eliminated in urine (Fukumoto et al., 2005; Plosker, 2010). The therapeutic plasma concentration of pilsicainide following oral administration is 0.2–0.9 μg/mL (Baselt, 2017a; Schulz et al., 2020), with fatal levels in blood reported within the range of 7.8–14.9 μg/mL (Hikiji et al., 2008; Fukasawa et al., 2018) (Table 2). Pilsicainide concentrations in the present case were all within this fatal range and markedly above the therapeutic range.

**TABLE 2:** Pilsicainide concentration in the body fluids associated with fatal intoxications reported in the scientific literature (µg/mL).

Specimen			Present case		Reference		
					Hikiji	Fukasawa	
					Hikiji et al. (2008)	Fukasawa et al. (2018)	
Blood							
Femoral venous blood			17.5			14.9	
Left heart chamber blood			26.6		8.0		
Right heart chamber blood			28.9		7.8		
Cardiac blood						22.6	
Bile			56.8				
Urine			136.9		8.3	150.0	
Cerebrospinal fluid			6.1				
Stomach contents			221.7			493.0	

Pilsicainide is recognized as a safe drug, but shows pro-arrhythmic effects in cases of intoxication. Serum pilsicainide levels have been reported to show a significant positive correlation with the electrocardiographic findings of PQ, QRS, and ST intervals and QTc prolongation (Horita et al., 2004; Koike et al., 2016). Pilsicainide induces tachyarrhythmias such as ventricular tachycardia, as a result of QTc and QRS prolongation (Horita et al., 2004; Kaneko et al., 2012), and bradyarrhythmias such as sinus pause and atrioventricular block (Toeda et al., 2000). As cases of sudden cardiac death have been reported for this drug (Nakatani et al., 2014), the pro-arrhythmic effects of pilsicainide were speculated to have contributed to this death.

The stomach contents showing high concentrations of pilsicainide, furosemide, and verapamil indicated that the patient had ingested pilsicainide along with other drugs. Since blood concentrations of verapamil were above the therapeutic range and those of furosemide were below the therapeutic range, the possibility of drug–drug interactions between verapamil and pilsicainide should be considered. Verapamil is known to inhibit the activity of CYP3A4 (Scheen, 2011), but a clinically relevant effect on pilsicainide metabolism by CYP2D6 would not be expected (Fujitani et al., 1997; Fukumoto et al., 2005). Although verapamil is known to inhibit P-glycoprotein (p-GP), this transporter’s contribution to the renal excretion of pilsicainide is negligible (Shiga et al., 2012). While there does not appear to be relevant pharmacokinetic drug–drug interactions due to CYP or p-GP inhibition, there is a potential pharmacodynamic drug–drug interaction between verapamil and pilsicainide. As verapamil itself induces pharmacological effects such as bradycardia, hypotension, and atrioventricular block (Baselt, 2017b), it compounds the risk of bradyarrhythmias when taken in combination with pilsicainide.

We also identified furosemide, warfarin, acetaminophen, ephedrine, and methylephedrine from the postmortem samples. Acetaminophen, ephedrine, and methylephedrine appeared to have been derived from over-the-counter cold remedies. As blood levels of those drugs were all below the therapeutic ranges, they were considered less likely to have contributed to this death.

Based on the autopsy findings, the results of the toxicological examinations, and the investigations by the authorities, we concluded that the cause of death was due to massive intake of pilsicainide, as its blood concentration was extremely high, with a possible contribution of verapamil to the lethal process.

## 3 Brief summary

Pilsicainide, a class IC anti-arrythmic agent, is prescribed for the treatment of supraventricular and ventricular tachyarrhythmias. Here, we report a fatal case of poisoning by pilsicainide. A high concentration of pilsicainide was detected in blood by liquid chromatography with tandem mass spectrometry. Although pilsicainide is recognized as a safe drug, it has pro-arrhythmic effects in case of an overdose.

## Data Availability

The original contributions presented in the study are included in the article; further inquiries can be directed to the corresponding author.
